# The Association Risk of Male Subfertility and Testicular Cancer: A Systematic Review

**DOI:** 10.1371/journal.pone.0005591

**Published:** 2009-05-18

**Authors:** Xiaoning Peng, Xiaomin Zeng, Sihua Peng, Defeng Deng, Jian Zhang

**Affiliations:** 1 Department of Internal Medicine, College of Medicine, Hunan Normal University, Changsha, People's Republic of China; 2 Department of Health Statistics and Epidemiology, School of Public Heath, Central South University, Changsha, People's Republic of China; 3 Department of Pathology, School of Medicine, Zhejiang University, Hangzhou, People's Republic of China; 4 Department of Cancer Biology, The University of Texas M. D. Anderson Cancer Center, Houston, Texas, United States of America; 5 Department of Biochemistry and Molecular Biology, College of Life Science, Hunan Normal University, Changsha, People's Republic of China; Johns Hopkins Bloomberg School of Public Health, United States of America

## Abstract

**Background:**

An association between male subfertility and an increased risk of testicular cancer has been proposed, but conflicting results of research on this topic have rendered this theory equivocal. To more precisely assess the association between subfertility and the risk of testicular cancer, we performed a systematic review of international epidemiologic evidence.

**Principal Findings:**

We searched the Medline database for records from January 1966 to March 2008 complemented with manual searches of the literature and then identified studies that met our inclusion criteria. Study design, sample size, exposure to subfertility and risk estimates of testicular cancer incidence were abstracted. Summary relative risks (RRs) with 95% confidence intervals (CIs) were calculated using the DerSimonian and Laird model. All statistical tests were two-sided. We identified seven case-control studies and two cohort studies published between 1987 and 2005. Analysis of the seven case-control studies that included 4,954 participants revealed an overall statistically significant association between subfertility and increased risk of testicular cancer (summary RR = 1.68, 95% CI: 1.22 to 2.31), without heterogeneity between studies (Q = 8.46, *P* heterogeneity = 0.21, *I* ^2^ statistics = 0.29). The association between subfertility and testicular cancer was somewhat stronger in the United States (summary RR = 1.75, 95% CI: 1.01 to 3.02) than it was in Europe (summary RR = 1.53, 95% CI: 1.22 to 1.92). The source of the control subjects had a statistically significant effect on the magnitude of the association (population-based summary—RR = 2.15, 95% CI: 1.11 to 4.17; hospital-based summary—RR = 1.56, 95% CI: 0.93 to 2.61). After excluding possible cryptorchidism, an important confounding factor, we also found a positive association between subfertility and increased risk of testicular cancer (summary RR = 1.59, 95% CI: 1.28 to 1.98). These results were consistent between studies conducted in the United States and in Europe (Q = 0.20, *P* heterogeneity = 0.66). Of the two cohort studies that reported standardized incidence ratios, both reported a statistically significant positive association between subfertility and increased risk of testicular cancer.

**Conclusions:**

Our findings support a relationship between subfertility and increased risk of testicular cancer and apply to the management of men with subfertility, and prevention and diagnosis of testicular cancer.

## Introduction

Testicular cancer is uncommon in most countries with an incidence that ranges from ∼1/100,000 to 10/100,000 and accounts for ∼1% of all cancers in men but ∼60% of all cancers in young males 15–35 years of age. Moreover, the incidence of testicular cancer has doubled in the last 20–40 years [1], [2]. The most common type of testicular cancer is testicular germ cell tumor (TGCT) and accounts for 95% of testicular cancers.

TGCTs are presumed to arise from a common precursor lesion, carcinoma in situ (CIS), which is found within the seminiferous tubules. TGCTs comprise two histologically distinct subtypes: seminomas and non-seminomas. The seminoma subtype consists of cells that resemble CIS but that are not constrained within the seminiferous tubules. The non-seminoma subtype represents tumors of mixed histology, including embryonal carcinoma, teratoma, and polyembryoma, choriocarcinoma, or yolk sac tumor [3].

At present, the etiology of testicular cancer is not well understood, but many risk factors—including cryptorchidism (a condition in which one or both testes fail to descend normally); inguinal hernia; contralateral testicular cancer; familial testicular cancer; testicular trauma; mumps orchitis; elevated testicular temperature; vasectomy; electromagnetic fields (EMF); and hormonal, prenatal, and occupational factors—have been implicated in this cancer's development in young adults [4]. The nature of these factors suggests that both genetic and environmental influences contribute to the development of testicular tumors. Until now, the most established factor associated with testicular cancer is cryptorchidism, which is associated with a 2- to 4-fold increase in the risk of testicular cancer but accounts for fewer than 10% of all cases [5].

Over the last 20–40 years, reports in the literature indicate a decrease in the quality of sperm and an increase in the frequency of testicular malignancies (especially seminoma) [6]–[8]. As many as 10 to 15% of all couples in western countries experience subfertility (the condition of being less than normally fertile though still capable of effecting fertilization), and in one third of these cases, the problem can be attributed to the male partner [6]–[8]. An association between the world-wide decrease in male fertility and testicular cancer has been suggested [9]–[17], although evidence for this type of association has been inconsistent [18]–[21], and the exact etiology of such an association remains debatable.

The potential association between subfertility and the testicular cancer has evoked a huge interest from clinicians, scientists, and the public [22]. Thus, to elucidate and to provide a quantitative assessment of the association between male subfertility and testicular cancer, we performed a systematic review of studies that evaluated such an association and our results indicated that male subfertility is significantly related to an increased risk for testicular cancer.

## Methods

### Search strategy

We conducted a literature search of the Medline database (records from January 1, 1966, through March 31, 2008) using three search terms, which were combined by the Boolean operator “and.” The first theme was (“Testicular Neoplasms”[Mesh] OR “testicular cancer” OR “testicular tumour”), the second theme was (“Infertility”[Mesh] OR “Infertility, Male”[Mesh] OR “subfertility”), and the third theme was (“Case-Control Studies”[Mesh] OR “case–control study” OR “Cohort Studies”[Mesh] OR “cohort study”). We sought additional eligible studies by conducting a manual search of reference lists from primary articles and relevant reviews. We identified epidemiologic studies conducted in humans from 1966 to 2008 that examined the association between subfertility and testicular cancer incidence. Two readers independently determined the eligibility of each article for inclusion in the meta-analysis. Any disagreements during the selection process were resolved by discussion with a third author.

### Selection

Eligible studies: 1) described a male population with an association between male subfertility and testicular cancer; 2) described a case-control study or cohort study; 3) for a case-control study, either provided an estimate of effect with odds ratio with 95% confidence interval (CI) or provided raw data from which and odds ratio that could be calculated; for a cohort study, provided an estimate of effect with rate ratio or standardized incidence ratio with 95% CI; 4) included a study population where the didagnosis of subfertility/fertility problem/infertility/low fertility prior to diagnosis of testicular cancer; and 5) were written in English.

Studies were excluded if no control group was included; if subjects had a history of cryptorchidism; if the indicators of male infertility, included offspring sex ratio, number of children, offspring twin rates, and offspring sibship size.These indicators are imperfect measures of male fertility, as they depend not the combined reproductive capacity of both the male and female partner. If duplicate publications from a single study were found,then we included only the article that provided more comprehensive information and was written in English.

### Validity assessment

Case-control studies were evaluated for quality using the quality assessment scale developed by Horwitz RI and colleagues [23], which include12 methodologic standards. For each study, compliance with each developed recommendation was rated according to the following possible scores: positive (+), negative (−), uncertain (±), and not applicable (NA) or not evaluable (NE). For cohort studies, we recorded the following quality indicators: approach to participant recruitment, length of follow-up, and consideration of confounding factors.

### Data abstraction

For each study, we abstracted the following data: 1) first author's name, year of publication, and country of the population studied; 2) study design; 3) characteristics of the study subjects (source of cases and controls, follow-up period for cohort studies); 4) number of exposed and unexposed subjects; 5) measures of outcome and exposure; 6) variables for which statistical adjustment was performed; 7) the relative risk (RR) or relative odds of testicular cancer being associated with subfertility and its 95% CI; and 8) history of cryptorchidism.

### Quantitative data synthesis

RR was used as the measure of effect of interest. We used the DerSimonian and Laird random-effects model to calculate a pooled RR and its corresponding 95% CI for studies that reported specific RRs [24], [25]. RRs from individual studies were transformed to their logarithms to stabilize the variances and to normalize the distributions. To assess for heterogeneity of RRs across studies, the Cochrane Q's statistic and the 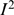 statistic were calculated [26]. The variance of the 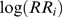 was derived from the CI provided in the study [27].

Sensitivity analyses were performed by omitting one study at a time and analyzing the remaining studies to detect whether the results were influenced excessively by any single study. The possibility of publication bias was assessed using visual inspection of a funnel plot [28], and both the Begg and Egger tests were also performed to assess the possibility of publication bias [29]. Furthermore, the Duval and Tweedie nonparametric trim-and-fill procedure was performed to further assess the possible effect of publication bias in our meta-analysis [30]. This method considers the possibility of hypothetical “missing” studies that might exist, imputes their RRs, and recalculates a pooled RR that incorporates the hypothetical missing studies as though they actually existed. Meta-analysis was performed using Microsoft Excel (Microsoft Corp, Seattle, Washington), SPSS11.5 (SPSS Corp, Chicago, Illinois), and Stata version 10.0 (Stata Corp, College Station, Texas). *P* values less than 0.05 were considered statistically significant, and all statistical tests were two-sided.

## Results

### Flow of included studies

**Figure 1:**
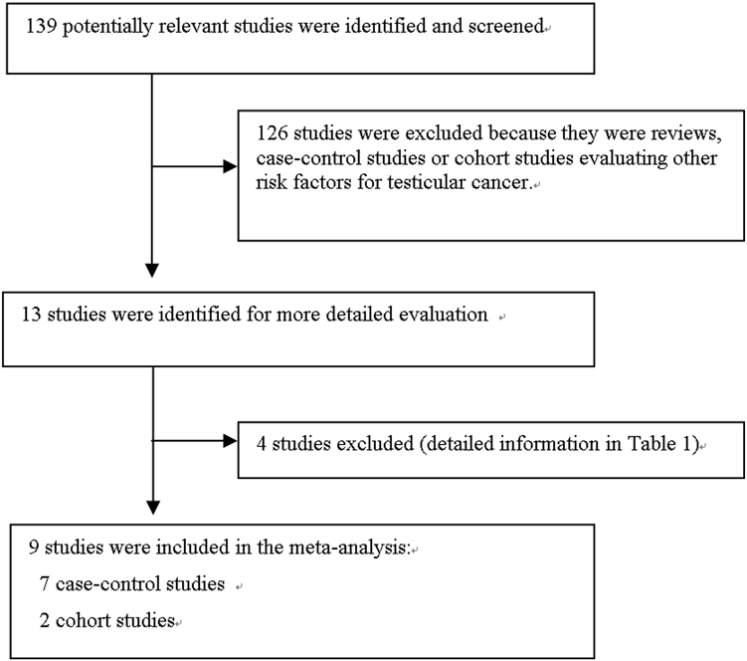
QUOROM flow diagram (flow of included studies).

From our review of abstracts, we initially selected 139 studies for a more detailed review (References S1); we excluded 126 studies because they were reviews, studies of other risk factors, or case reports. From the remaining 13 studies, we excluded four because the indicator to measure the male fertility problem was one of the following: sex ratio, twin rates, and sibship size of offspring—or because the population diagnosed with subfertility had a history of cryptorchidism (Table 1). After review, nine studies were included in the meta-analysis. Of the nine studies, seven were case-control studies—four from the United States and three from Europe; two of the nine were cohort studies—one from the United States and one from Europe (Fig. 1).

**Table 1:** Reasons for four studies excluded from the meta-analysis.

First author	Year (Ref. No.)	Region	Design	Reasons for Exclusion
Richiardi L	2004 (15)	Europe	Cohort	Included participants with a history of cryptorchidism; used offspring twinning rates to define infertility
Fossa SD	2000 (16)	Europe	Cohort	Used number of children to define infertility
Jacobsen R	2000 (17)	Europe	Cohort	Used offspring sex-ratio to define infertility
Henderson BE	1979 (21)	USA	Case-control	Used offspring sibship size to define infertility

### Study characteristics

The characteristics of the included studies are listed in Table 2 (for case-control studies) and Table 3 (for cohort studies). The nine studies that we included in the meta-analysis were published between 1987 and 2005. The studies about subfertility were registries or interviews and questionnaires. Seven case-control studies used odds ratios and the 95% CIs as the measure of RR [9]–[12], [18]–[20] and enrolled a total of 4,954 participants. The total subfertility case number was 2,099 (range of case subjects: 140 to 514), and the total number of control subjects was 2,855 (range of control subjects: 135 to 720). Case subjects were all identified in the cancer registry, with three hospital-based control subjects [12], [18], [20] and four population-based control subjects [9]–[11], [19]. Two cohort studies used standardized incidence ratios and the 95% CIs as the measure of RR [13], [14] and had enrolled a total of 36,289 participants, with a mean follow-up period ranging from 6 to 20 years. Five studies were conducted in the United States (four case-control studies and one cohort study) and four in Europe (three case-control studies and one cohort study).

**Table 2:** Characteristics of seven case-control studies of subfertility and testicular cancer incidence.

First Author	Year (Ref. No.)	Country	Excluded Cryptorchidism	No. of Cases (Source of Cases)/No. of Controls (Selection Method)	Estimated Odds Ratios (95% CI)	Controlled Variables
Baker JA	2005 (9)	USA	NA*	201 (Cancer registry)/204 (Population controls matched by age and area of residence)	9.47 (1.19–75.2)	Age, neighborhood
Møller H	1999 (10)	Europe (Danish)	Yes	514 (Cancer registry)/720 (Population controls matched by age)	1.42 (1.09–1.85)	Age
Doria-Rose VP	2005 (11)	USA	Yes	329 (Cancer registry)/672 (Population controls matched by age, race, language and area of residence)	2.51 (1.07–5.86)	Age
Swerdlow AJ	1989 (12)	Europe (England)	Yes	178 (Cancer registry)/315 (Hospital patients matched by treatment center, area of residence and duration of follow-up)	1.73 (1.09–2.74)	Area of hospital
Forman D PM	1994 (18)	Europe (England and Wales)	Yes	775 (Cancer registry)/794 (Hospital patients matched by age, register hospital or center, enrollment date and area of residence)	2.66 (0.94–7.54)	Age, area of residence, and register-time
Haughey BP	1989 (19)	USA	Yes	250 (Cancer registry)/250 (Population controls matched by age and neighborhood of residence)	4.77 (0.40–52.50)	Age, neighborhood
Brown LM	1987 (20)	USA	NA	140 (Cancer registry)/135 (Hospital patients without cancer matched by treatment hospital, age, race, vital status, year of diagnosis, and hospital of diagnosis)	0.89 (0.40–1.96)	Age, race, vital status and year of diagnosis

* NA, not available. CI, confidence interval.

**Table 3:** Characteristics of two cohort studies of subfertility and testicular cancer incidence.

First author	Year (Ref. No)	Country	Excluded Cryptorchidism	Study Population	Follow-up Period	Estimated Standardized Incidence Ratios (95% CI)	Controlled Variables
Jacobsen R	2000 (13)	Europe (Danish)	NA*	Exposed group: 32,442 subjects with a semen analysis (No. of cases: 89)	1963–1995	1.60 (1.30–1.90)	Age
				Comparison group: Danish population			
Raman JD	2005 (14)	USA	Yes	Exposed group: 3,847 subjects with abnormal semen analysis (No. of cases: 8)	1990–2000	18.30 (18.00–18.80)	Age, race
				Comparison group: American population			

* NA, not available.

### Quality assessment

The quality assessment score for the included seven case-control studies is presented in Table 4. The criteria “Predetermined method of selection”, “Specification of the causal agent”, “Avoidance of constrained cases”, and “Avoidance of constrained controls” were scored as positive in all studies. “Unbiased data collection” was scored as positive in one study [12], negative in one study [10], and uncertain in four studies [9], [19]. “Anamnestic equivalence” was scored as positive in two studies [9], [12], and “Equal demographic susceptibility” was scored as positive in six studies and uncertain in one study [9], [19]. “Equal clinical susceptibility” was scored as positive in five studies and NA/NE in two studies [9], [19]. “Avoidance of Berkson's bias” was scored as positive in four studies [9], [19] and negative in three studies [9], [19]. “Equal diagnostic examination” was scored as positive in two studies [9], [19] and NA/NE in five studies. Table 5 presents corresponding quality criteria for the two cohort studies that were included in our review, and these two studies met many of the measured quality criteria [9], [19].

**Table 4:** Compliance with the 12 methodologic criteria in seven case-control studies.

First Author	Baker JA	Møller H	Doria-Rose VP	Swerdlow AJ	Forman D PM	Haughey BP	Brown LM
Year (Ref. No.)	2005 (9)	1999 (10)	2005 (11)	1989 (12)	1994 (18)	1989 (19)	1987 (20)
Predetermined method	+	+	+	+	+	+	+
Specification of the agent	+	+	+	+	+	+	+
Unbiased data collection	±	−	±	+	±	±	△
Anamnestic eauivalence	+	△	±	+	±	±	±
Avoidance of constrained cases	+	+	+	+	+	+	+
Avoidance of constrained controls	+	+	+	+	+	+	+
Equal diagnostic examination	△	△	△	+	△	△	+
Equal diagnostic surveillance	△	△	△	△	△	△	△
Equal demographic susceptibility	+	+	+	±	+	+	+
Equal clinical susceptibility	△	+	+	+	+	+	△
Avoidance of protopathic bias	△	△	△	△	△	△	△
Community control for Berkson's bias	+	+	+	−	−	+	−

+, positive; −, negative; ±, uncertain; △, not applicable or not evaluable.

**Table 5:** Quality indicators for cohort studies.

First author	Year (Ref. No)	Same mode of inclusion for intervention and control group	Enough F/U duration	Report of loss of F/U	Adjusted analysis for confounding variables	Mode of participants selection described	Potential important baseline differences	Sample size prespecified	Report of important baseline characteristics modification during F/U
Jacobsen R	2000 (13)	Yes	Yes	No	Yes	Yes	No	Yes	No
Raman JD	2005 (14)	Yes	Yes	No	Yes	Yes	Yes	Yes	Yes

### Quantitative data synthesis

**Figure 2:**
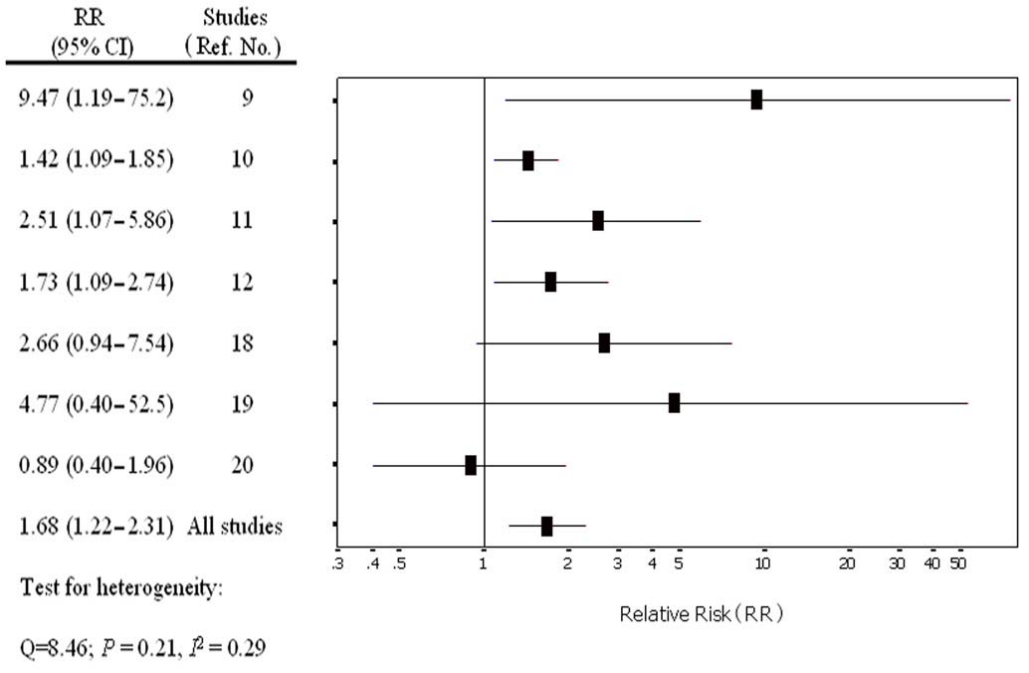
Association between subfertility and testicular cancer incidence in the seven case-control studies.

Individual study results and the overall summary results for the seven case-controlled studies on the association between subfertility and testicular cancer incidence are shown in Table 6. By pooling the results of these seven studies [9]–[12], [18]–[20], we found that subfertility was associated with an increased risk of testicular cancer (summary RR = 1.68, 95% CI: 1.22 to 2.31)(Fig. 2). In a sensitivity analysis in which one study at a time was excluded and the rest were analyzed, we detected a statistically significant positive association between subfertility and testicular cancer incidence (range of summary RRs = 1.56 to 1.92 and all the lower limit of the 95% CIs>1.0).We then conducted subgroup meta-analyses by the source of control subjects, geographical area, and possible presence of cryptorchidism. Analysis of four studies that included population-based control subjects showed a positive association (summary RR = 2.15, 95% CI = 1.11 to 4.17). Analysis of three studies with hospital-based subjects [12], [18], [20], did not support an association between subfertility and higher incidence of testicular cancer (summary RR = 1.56, 95% CI = 0.93 to 2.61).

**Table 6:** Summary relative risk (RR) estimates and 95% confidence intervals (CIs) for seven case-control studies of the association between subfertility and testicular cancer incidence.

Subgroup	Studies (No.)	Summary RR (95% CI)	Between Studies		
			Q	*P* Heterogeneity	*I* ^2^ Statistics
Case-control studies	7	1.68 (1.22 to 2.3)	8.46	0.21	0.29
Population-based	4	2.15 (1.11 to 4.17)	5.36	0.15	0.44
Hospital-based	3	1.56 (0.93 to 2.61)	3.09	0.21	0.35
Region			0.20*	0.66	
United States	4	1.75 (1.01 to 3.02)	6.61	0.09	0.55
Europe	3	1.53 (1.22 to 1.92)	1.66	0.44	0.20
Excluded cryptorchidism	5	1.59 (1.28 to 1.98)	3.63	0.46	0.10

* for between subgroup.

We found that a positive association between subfertility and testicular cancer by region (the United States and Europe), with a somewhat stronger association in the United States [9], [11], [19]–[20] (summary RR = 1.75, 95% CI = 1.01 to 3.02) than in Europe [10], [12], [18] (summary RR = 1.53, 95% CI = 1.22 to 1.92).

As a strong risk factor for both testicular cancer and subfertility, cryptorchidism is a potentially important confounder of the positive association between subfertility and testicular cancer. We analyzed five studies [10]–[12], [18]–[19] that excluded participants with a history of cryptorchidism, and found a positive association between subfertility and testicular cancer (summary RR = 1.59, 95% CI = 1.28 to 1.98).

Both cohort studies identified (one conducted in the United States and the other in Europe) [13], [14] reported a standardized incidence ratios and a statistically significant positive association between subfertility and testicular cancer (United States population RR = 18.3, 95% CI = 18.0 to 18.8; Danish population RR = 1.6, 95% CI = 1.3 to 1.9).

### Publication bias

**Figure 3:**
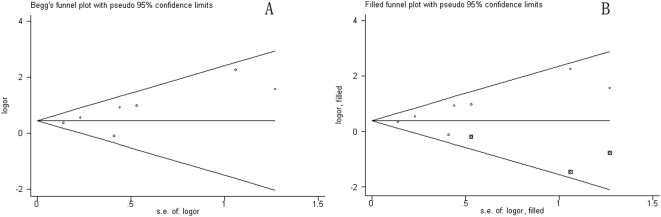
Funnel plots with and without trim and fill.

The funnel plot appears asymmetric (Fig. 3A), suggesting publication bias—although the Begg test and Egger test were not statistically significant (*P* = 0.23 and 0.11, respectively). Therefore, we performed a sensitivity analysis by using the trim-and-fill method [30], which conservatively imputes hypothetical negative unpublished studies to mirror the positive studies that cause funnel plot asymmetry. The imputed studies produce a symmetrical funnel plot (Figure 3B). The pooled analysis incorporating the hypothetical studies continued to show a statistically significant association between testicular cancer and subfertility (with adjusted RR = 1.51, 95% CI: 1.08–2.12; *P*<0.017).

## Discussion

Discrepancies among previous studies investigating the relationship of subfertility with testicular cancer risk may be attributable to small sample sizes that resulted in insufficient statistical power to detect some relationships in the individual studies. To address this uncertainty, we used rigorous meta-analytic techniques to quantitatively assess the association more precisely. The results of our meta-analysis support the association of subfertility with subsequent risk for testicular cancer.

By pooling data from seven case-control studies meeting our inclusion criteria, we demonstrated that individuals with subfertility have an overall increased RR of developing testicular cancer compared with control individuals; the results suggested a consistent positive association between subfertility and testicular cancer for studies carried out in the United States and in Europe, for studies of population-based and hospital-based control subjects, and for studies that completely excluded cryptorchidism. Two cohort studies could not be made into a pooled analysis because standardized incidence ratios were used as the measure of effect of interest; however, both studies reported a statistically significant positive association between subfertility and testicular cancer, with a 1.6-and an 18.3-fold increased risk of testicular cancer among subfertility patients, respectively.

Like all meta-analyses, this study had some limitations. First, the definition of subfertility in this systematic review is a somewhat comprehensive definition, and we included the studies that reported on men who had been diagnosed with subfertility/fertility problem/infertility/low fertility due to difficulty getting a partner pregnant or to a sperm problem prior to a diagnosis of testicular cancer. Second, the quality of case-control individual studies was inconsistent as is shown in Table 4, with a lack of unbiased data collection, anamnestic equivalence, equal diagnostic examination, equal clinical susceptibility, and “community control” for Berkson's bias for some studies. Third, we only found two cohort studies that reported standardized incidence ratios and satisfied our inclusion criteria, and the data from these two cohort studies could not be pooled with data from case-control studies. In addition, we only found five studies that reported no history of cryptorchidism and restricted our search strategy to include articles in English only.

The results we obtained from the funnel plot analysis were consistent with publication bias, that is, the presence of unpublished negative studies. Because of this, we undertook a sensitivity analysis using the trim-and-fill method. The trim-and-fill sensitivity analysis did not change direction of the results, although the strength of the association was somewhat weakened, indicating that the association is not an artifact of unpublished negative studies. Nevertheless, that possibility is not fully excluded by this method. Therefore, further studies are needed to supplement the results of this meta-analysis.

A relationship between subfertility and increased risk of testicular cancer is epidemiologically plausible. First, it has been suggested that during the past four decades human sperm counts have declined and the incidence of testicular cancer has increased [31]–[33]. Second, the incidence rate of testicular cancer in infertile men (∼0.5–1%) is much higher than that of the general population (0.001%∼0.01%), and men who present with subfertility are of a similar age as men with the highest prevalence of testicular cancer [14], [34], [35]. Third, after excluding the common risk factors for testicular cancer and subfertility, such as the history of cryptorchidism and chromosomal aberrations, men with infertility also have an increased risk of developing testicular cancer [11], [14], [22].

A relationship between subfertility and increased risk of testicular cancer is biologically plausible. First, fetal and neonatal life are important in the development of reproductive disorders, and some studies have shown that in utero exposure to excess environmental estrogens (EES) is a risk factor for subfertility and testicular cancer [15], [36]–[38]. Though the mechanism that mediates these effects is not well understood, some investigators propose that EES not only interferes in the normal testis endocrine directly but also disrupts the hypothalamic-pituitary-testis axis, resulting in abnormal function of sertoli cells; this disruption impairs germ cell differentiation and the germ cells are subsequently transformed to develop into carcinoma in situ (CIS) [4], [39]. Estimates predict that CIS will develop into testicular cancer within 5 years of diagnosis in half of all cases, and all patients who harbor CIS cells at puberty will eventually develop testicular cancer [4].

Second, genetic defects provide robust evidence in favor of a relationship between subfertility and increased risk of testicular cancer. Brothers of men with testicular cancer have lower fertility and a higher risk of having testicular cancer than age matched controls [16]. The “gr/gr” (carries a number of genes specifically involved in male germ cell development) deletion in the Y chromosome has been found to be associated with subfertility and TGCT [40]. Youngren et al. found that the Ter mutation in the dead end gene (Dnd1) causes primordial germ cell (PGCs) loss and TGCTs, and loss of PGCs precedes development of embryonal carcinoma cells in 129-Ter/Ter mouse embryos [41]. Therefore, inactivation of Dnd1 expression is implicated as the causal event that drives PGCs to exit the germ line and transform to embryonal carcinoma cells and TGCTs in 129-Ter/Ter mice [41].

Our findings provide evidence for a role of subfertility in carcinogenesis of testicular cancer. Our study numbers were limited and all of the involved studies were conducted in the United States and Europ. In addition, selection and recall biases may affect the association between male subfertility and the subsequent development of testicular cancer. More and larger case control studies or prospective studies with multi-regional and multi-institutional cohorts of men presenting for subfertility or infertility care are needed for a clearer determination of the association of subfertility with increased risk of testicular cancer.

Testicular cancer has a complex etiology; environmental factors and genetic mutations both contribute to the increased risk of developing testicular cancer. Therefore, future studies will take up the exciting challenge to elucidate the mechanism of the interaction between environmental factors and genetic mutations (such as how the estrogen regulates the related gene of germ cell development) that influences the spermatogenesis and carcinogenesis of germ cells.

## Supporting Information

References S1 (0.07 MB DOC)
