# Supplementary material for: The Association Risk of Male Subfertility and Testicular Cancer: A Systematic Review
Source: PLoS One. 2009 May 18;4(5):e5591. doi: 10.1371/journal.pone.0005591 (PMC2680046; doi:10.1371/journal.pone.0005591)
Supplement: References S1 — (0.07 MB DOC) [file pone.0005591.s001.doc]

**References S1**

**Article search results:**

1. Ou S-M, Lee S-S, Tang S-H, Wu S-T, Wu C-J, et al. (2007) Testicular microlithiasis in Taiwanese men. Archives of andrology 53: 339-344.

2. Izegbu MC, Ojo MO, Shittu LAJ (2005) Clinico-pathological patterns of testicular malignancies in Ilorin, Nigeria--a report of 8 cases. Journal of cancer research and therapeutics 1: 229-231.

3. Suzuki K, Matuzaki J, Hattori Y, Saito K, Noguchi K, et al. (2007) [Semen cryopreservation for patients with malignant or non-malignant disease: our 14-year experience]. Hinyokika kiyo Acta urologica Japonica 53: 539-544. In Japanese.

4. Olesen IA, Hoei-Hansen CE, Skakkebaek NE, Petersen JH, Rajpert-De Meyts E, et al. (2007) Testicular carcinoma in situ in subfertile Danish men. International journal of andrology 30: 406-411; discussion 412.

5. Edelstein A, Yavetz H, Kleiman SE, Botchan A, Hauser R, et al. (2008) Deoxyribonucleic acid-damaged sperm in cryopreserved-thawed specimens from cancer patients and healthy men. Fertility and sterility 90: 205-208.

6. Choi J, Koh E, Matsui F, Sugimoto K, Suzuki H, et al. (2008) Study of azoospermia factor-a deletion caused by homologous recombination between the human endogenous retroviral elements and population-specific alleles in Japanese infertile males. Fertility and sterility 89: 1177-1182.

7. Kanto S, Takahashi K, Maehara I, Fukuzaki A, Kyono K, et al. (2007) Incidental testicular cancers that subsequently developed in oligozoospermic and azoospermic patients: report of three cases. Fertility and sterility 88: 1374-1376.

8. Wang Y, Barthold J, Kanetsky PA, Casalunovo T, Pearson E, et al. (2007) Allelic variants in HOX genes in cryptorchidism. Birth defects research Part A, Clinical and molecular teratology 79: 269-275.

9. Connolly SS, D'Arcy FT, Gough N, McCarthy P, Bredin HC, et al. (2006) Carefully selected intratesticular lesions can be safely managed with serial ultrasonography. BJU international 98: 1005-1007; discussion 1007.

10. Garolla A, Pizzato C, Ferlin A, Carli MO, Selice R, et al. (2006) Progress in the development of childhood cancer therapy. Reproductive toxicology (Elmsford, N Y ) 22: 126-132.

11. Zorn B, Virant-Klun I, Stanovnik M, Drobnic S, Meden-Vrtovec H (2006) Intracytoplasmic sperm injection by testicular sperm in patients with aspermia or azoospermia after cancer treatment. International journal of andrology 29: 521-527.

12. Granados Loarca EA, Esau Ortega S (2005) [Is necessary to practice orchiectomy in patients with post-puberal maldescended testes?]. Actas urologicas espanolas 29: 969-973. In Spanish.

13. Haddad O, Leroy X, Lemaitre L, Biserte J, Rigot J-M (2005) [Infertility and testicular tumour based on a series of 25 patients]. Progres en urologie : journal de l'Association francaise d'urologie et de la Societe francaise d'urologie 15: 1096-1100. In French.

14. Richiardi L, Akre O (2005) Fertility among brothers of patients with testicular cancer. Cancer epidemiology, biomarkers & prevention : a publication of the American Association for Cancer Research, cosponsored by the American Society of Preventive Oncology 14: 2557-2562.

15. Raman JD, Nobert CF, Goldstein M (2005) Increased incidence of testicular cancer in men presenting with infertility and abnormal semen analysis. The Journal of urology 174: 1819-1822; discussion 1822.

16. Carmignani L, Morabito A, Gadda F, Bozzini G, Rocco F, et al. (2005) Prognostic parameters in adult impalpable ultrasonographic lesions of the testicle. The Journal of urology 174: 1035-1038.

17. Doria-Rose VP, Biggs ML, Weiss NS (2005) Subfertility and the risk of testicular germ cell tumors (United States). Cancer causes & control : CCC 16: 651-656.

18. Aschim EL, Giwercman A, Stahl O, Eberhard J, Cwikiel M, et al. (2005) The RsaI polymorphism in the estrogen receptor-beta gene is associated with male infertility. The Journal of clinical endocrinology and metabolism 90: 5343-5348.

19. Baker JA, Buck GM, Vena JE, Moysich KB (2005) Fertility patterns prior to testicular cancer diagnosis. Cancer causes & control : CCC 16: 295-299.

20. Boehmer D, Badakhshi H, Kuschke W, Bohsung J, Budach V (2005) Testicular dose in prostate cancer radiotherapy: impact on impairment of fertility and hormonal function. Strahlentherapie und Onkologie : Organ der Deutschen Rontgengesellschaft [et al] 181: 179-184.

21. Bahadur G, Ozturk O, Muneer A, Wafa R, Ashraf A, et al. (2005) Semen quality before and after gonadotoxic treatment. Human reproduction (Oxford, England) 20: 774-781.

22. Nalesnik JG, Sabanegh ES, Eng TY, Buchholz TA (2004) Fertility in men after treatment for stage 1 and 2A seminoma. American journal of clinical oncology 27: 584-588.

23. Hosalkar HS, Henderson KM, Weiss A, Donthineni R, Lackman RD (2004) Chemotherapy for bone sarcoma does not affect fertility rates or childbirth. Clinical orthopaedics and related research: 256-260.

24. Jedrzejczak P, Taszarek-Hauke G, Korman M, Kopaczynski P, Pawelczyk L (2004) [The sperm quality in young patients before cancer therapy]. Przegla d lekarski 61: 141-145. In Polish.

25. Tal R, Holland R, Belenky A, Konichezky M, Baniel J (2004) Incidental testicular tumors in infertile men. Fertility and sterility 82: 469-471.

26. Kanto S, Hiramatsu M, Suzuki K, Ishidoya S, Saito H, et al. (2004) Risk factors in past histories and familial episodes related to development of testicular germ cell tumor. International journal of urology : official journal of the Japanese Urological Association 11: 640-646.

27. Bordallo MAN, Guimaraes MM, Pessoa CHCN, Carrico MK, Dimetz T, et al. (2004) Decreased serum inhibin B/FSH ratio as a marker of Sertoli cell function in male survivors after chemotherapy in childhood and adolescence. Journal of pediatric endocrinology & metabolism : JPEM 17: 879-887.

28. Mirimanoff RO (2003) [Radiotherapy of testicular seminoma: changes over the past 10 years]. Cancer radiotherapie : journal de la Societe francaise de radiotherapie oncologique 7 Suppl 1: 70s-77s. In French.

29. Pelliccione F, Cordeschi G, Giuliani V, D'Abrizio P, Necozione S, et al. (2004) The contractile wall of the caput epididymidis in men affected by congenital or postinflammatory obstructive azoospermia. Journal of andrology 25: 417-425.

30. Dieckmann KP, Pichlmeier U (2004) Clinical epidemiology of testicular germ cell tumors. World journal of urology 22: 2-14.

31. Huyghe E, Matsuda T, Daudin M, Chevreau C, Bachaud J-M, et al. (2004) Fertility after testicular cancer treatments: results of a large multicenter study. Cancer 100: 732-737.

32. de Gouveia Brazao CA, Pierik FH, Oosterhuis JW, Dohle GR, Looijenga LHJ, et al. (2004) Bilateral testicular microlithiasis predicts the presence of the precursor of testicular germ cell tumors in subfertile men. The Journal of urology 171: 158-160.

33. Steiner H, Holtl L, Maneschg C, Berger AP, Rogatsch H, et al. (2003) Frozen section analysis-guided organ-sparing approach in testicular tumors: technique, feasibility, and long-term results. Urology 62: 508-513.

34. Gudbjartsson T, Magnusson K, Bergthorsson J, Barkardottir RB, Agnarsson BA, et al. (2003) A population-based analysis of increased incidence and improved survival of testicular cancer patients in Iceland. Scandinavian journal of urology and nephrology 37: 292-298.

35. Santoni R, Barbera F, Bertoni F, De Stefani A, Livi L, et al. (2003) Stage I seminoma of the testis: a bi-institutional retrospective analysis of patients treated with radiation therapy only. BJU international 92: 47-52; discussion 52.

36. Pamenter B, De Bono JS, Brown IL, Nandini M, Kaye SB, et al. (2003) Bilateral testicular cancer: a preventable problem? Experience from a large cancer centre. BJU international 92: 43-46.

37. Panidis D, Rousso D, Matalliotakis I, Kourtis A, Mavromatidis G, et al. (2003) Do characteristic spermatozoal morphological abnormalities exist in patients who have undergone unilateral orchiectomy and preventive radiotherapy? International journal of fertility and women's medicine 48: 83-87.

38. Meseguer M, Garrido N, Remohi J, Pellicer A, Simon C, et al. (2003) Testicular sperm extraction (TESE) and ICSI in patients with permanent azoospermia after chemotherapy. Human reproduction (Oxford, England) 18: 1281-1285.

39. Crha I, Ventruba P, Petrenko M, Zakova J, Visnova H, et al. (2002) [Cryopreservation of sperm before neoplasm therapy--7 years' experience]. Ceska gynekologie / Ceska lekarska spolecnost J Ev Purkyne 67: 324-328. In Czech.

40. Demkow T, Faundez R, Kamoda J (2002) [Sperm evaluation in testicular cancer patients before and after chemotherapy]. Ginekologia polska 73: 845-852. In Polish.

41. Thomson AB, Campbell AJ, Irvine DC, Anderson RA, Kelnar CJH, et al. (2002) Semen quality and spermatozoal DNA integrity in survivors of childhood cancer: a case-control study. Lancet 360: 361-367.

42. Hyer S, Vini L, O'Connell M, Pratt B, Harmer C (2002) Testicular dose and fertility in men following I(131) therapy for thyroid cancer. Clinical endocrinology 56: 755-758.

43. Damani MN, Master V, Meng MV, Burgess C, Turek P, et al. (2002) Postchemotherapy ejaculatory azoospermia: fatherhood with sperm from testis tissue with intracytoplasmic sperm injection. Journal of clinical oncology : official journal of the American Society of Clinical Oncology 20: 930-936.

44. Joly F, Heron JF, Kalusinski L, Bottet P, Brune D, et al. (2002) Quality of life in long-term survivors of testicular cancer: a population-based case-control study. Journal of clinical oncology : official journal of the American Society of Clinical Oncology 20: 73-80.

45. von Eckardstein S, Tsakmakidis G, Kamischke A, Rolf C, Nieschlag E (2001) Sonographic testicular microlithiasis as an indicator of premalignant conditions in normal and infertile men. Journal of andrology 22: 818-824.

46. Kolettis PN, Sabanegh ES (2001) Significant medical pathology discovered during a male infertility evaluation. The Journal of urology 166: 178-180.

47. Baniel J, Sella A (2001) Sperm extraction at orchiectomy for testis cancer. Fertility and sterility 75: 260-262.

48. Tournaye H (2000) Storing reproduction for oncological patients: some points for discussion. Molecular and cellular endocrinology 169: 133-136.

49. Jacobsen R, Bostofte E, Engholm G, Hansen J, Olsen JH, et al. (2000) Risk of testicular cancer in men with abnormal semen characteristics: cohort study. BMJ (Clinical research ed ) 321: 789-792.

50. Moller H, Skakkebaek NE (1999) [Occurrence of testicular cancer in subfertile men. A case-control study]. Ugeskrift for laeger 161: 6490-6492. In Danish.

51. Salanova M, Gandini L, Lenzi A, Chiarenza C, Filippini A, et al. (1999) Is hyperdiploidy of immature ejaculated germ cells predictive of testis malignancy? A comparative study in healthy normozoospermic, infertile, and testis tumor suffering subjects. Laboratory investigation; a journal of technical methods and pathology 79: 1127-1135.

52. Kalfon A, Abram F, Kirsch-Noir F, Tchovelidze C, Arvis G (1999) [Leydig cell testicular tumors. A series of 10 observations]. Progres en urologie : journal de l'Association francaise d'urologie et de la Societe francaise d'urologie 9: 299-304. In French.

53. Hartmann JT, Albrecht C, Schmoll HJ, Kuczyk MA, Kollmannsberger C, et al. (1999) Long-term effects on sexual function and fertility after treatment of testicular cancer. British journal of cancer 80: 801-807.

54. Moller H, Skakkebaek NE (1999) Risk of testicular cancer in subfertile men: case-control study. BMJ (Clinical research ed ) 318: 559-562.

55. Marmor D, Izard V, Schahmaneche D, Benoit G, Jardin A (1998) [Is today's man really less fertile?]. Presse medicale (Paris, France : 1983) 27: 1484-1490. In French.

56. Rogers E, Teahan S, Gallagher H, Butler MR, Grainger R, et al. (1998) The role of orchiectomy in the management of postpubertal cryptorchidism. The Journal of urology 159: 851-854.

57. Muller HL, Klinkhammer-Schalke M, Seelbach-Gobel B, Hartmann AA, Kuhl J (1996) Gonadal function of young adults after therapy of malignancies during childhood or adolescence. European journal of pediatrics 155: 763-769.

58. Moutel G, Corviole K, Delepine B, Alnot MO, Melin MC, et al. (1996) [Evolution of the practice of sperm cryopreservation and fate of the preserved specimens : retrospective study of testicular cancer]. Contraception, fertilite, sexualite (1992) 24: 589-595. In French.

59. Heimdal K, Andersen TI, Skrede M, Fossa SD, Berg K, et al. (1995) Association studies of estrogen receptor polymorphisms in a Norwegian testicular cancer population. Cancer epidemiology, biomarkers & prevention : a publication of the American Association for Cancer Research, cosponsored by the American Society of Preventive Oncology 4: 123-126.

60. Honig SC, Lipshultz LI, Jarow J (1994) Significant medical pathology uncovered by a comprehensive male infertility evaluation. Fertility and sterility 62: 1028-1034.

61. Bettocchi C, Coker CB, Deacon J, Parkinson C, Pryor JP (1994) A review of testicular intratubular germ cell neoplasia in infertile men. Journal of andrology 15 Suppl: 14S-16S.

62. Centola GM, Keller JW, Henzler M, Rubin P (1994) Effect of low-dose testicular irradiation on sperm count and fertility in patients with testicular seminoma. Journal of andrology 15: 608-613.

63. Malas S, Levin V, Sur RK, Donde B, Krawitz HE, et al. (1994) Fertility in patients treated with radiotherapy following orchidectomy for testicular seminoma. Clinical oncology (Royal College of Radiologists (Great Britain)) 6: 377-380.

64. Thomas DB, Jimenez LM, McTiernan A, Rosenblatt K, Stalsberg H, et al. (1992) Breast cancer in men: risk factors with hormonal implications. American journal of epidemiology 135: 734-748.

65. Pryor JP, Hendry WF (1991) Ejaculatory duct obstruction in subfertile males: analysis of 87 patients. Fertility and sterility 56: 725-730.

66. Talati J, Sheikh H (1991) Sertoli cell only syndrome (SECOS): lessons from case studies. JPMA The Journal of the Pakistan Medical Association 41: 219-223.

67. Rieker PP, Fitzgerald EM, Kalish LA, Richie JP, Lederman GS, et al. (1989) Psychosocial factors, curative therapies, and behavioral outcomes. A comparison of testis cancer survivors and a control group of healthy men. Cancer 64: 2399-2407.

68. Swerdlow AJ, Huttly SR, Smith PG (1989) Testis cancer: post-natal hormonal factors, sexual behaviour and fertility. International journal of cancer Journal international du cancer 43: 549-553.

69. Carroll PR, Whitmore WF, Richardson M, Bajorunas D, Herr HW, et al. (1987) Testicular failure in patients with extragonadal germ cell tumors. Cancer 60: 108-113.

70. Fossa SD, Almaas B, Jetne V, Bjerkedal T (1986) Paternity after irradiation for testicular cancer. Acta radiologica Oncology 25: 33-36.

71. Rhodes EA, Hoffman DJ, Kaempfer SH (1985) Ten years of experience with semen cryopreservation by cancer patients: follow-up and clinical considerations. Fertility and sterility 44: 512-516.

72. Johnson DH, Hainsworth JD, Linde RB, Greco FA (1984) Testicular function following combination chemotherapy with cis-platin, vinblastine, and bleomycin. Medical and pediatric oncology 12: 233-238.

73. Nicholls DP, Anderson DC (1982) Clinical aspects of androgen deficiency in men. Andrologia 14: 379-388.

74. O'Flaherty C, Vaisheva F, Hales BF, Chan P, Robaire B (2008) Characterization of sperm chromatin quality in testicular cancer and Hodgkin's lymphoma patients prior to chemotherapy. Human reproduction (Oxford, England) 23: 1044-1052.

75. Crha I, Ventruba P, Zakova J, Kubesova B, Jarkovsky J, et al. (2007) [Sperm banking before gonadotoxic treatment--11-years experience]. Ceska gynekologie / Ceska lekarska spolecnost J Ev Purkyne 72: 320-326. In Czech.

76. Giannarini G, Mogorovich A, Menchini Fabris F, Morelli G, De Maria M, et al. (2007) Long-term followup after elective testis sparing surgery for Leydig cell tumors: a single center experience. The Journal of urology 178: 872-876; quiz 1129.

77. Dieckmann K-P, Linke J, Pichlmeier U, Kulejewski M, Loy V (2007) Spermatogenesis in the contralateral testis of patients with testicular germ cell cancer: histological evaluation of testicular biopsies and a comparison with healthy males. BJU international 99: 1079-1085.

78. Efstathiou E, Logothetis CJ (2006) Review of late complications of treatment and late relapse in testicular cancer. Journal of the National Comprehensive Cancer Network : JNCCN 4: 1059-1070.

79. Gandini L, Sgro P, Lombardo F, Paoli D, Culasso F, et al. (2006) Effect of chemo- or radiotherapy on sperm parameters of testicular cancer patients. Human reproduction (Oxford, England) 21: 2882-2889.

80. Sato Y, Yoshida K, Shinka T, Nozawa S, Nakahori Y, et al. (2006) Altered expression pattern of heat shock transcription factor, Y chromosome (HSFY) may be related to altered differentiation of spermatogenic cells in testes with deteriorated spermatogenesis. Fertility and sterility 86: 612-618.

81. Thorup J, Cortes D, Petersen BL (2006) The incidence of bilateral cryptorchidism is increased and the fertility potential is reduced in sons born to mothers who have smoked during pregnancy. The Journal of urology 176: 734-737.

82. Serter S, Gumus B, Unlu M, Tuncyurek O, Tarhan S, et al. (2006) Prevalence of testicular microlithiasis in an asymptomatic population. Scandinavian journal of urology and nephrology 40: 212-214.

83. Lundin KB, Nordenskjold A, Giwercman A, Giwercman YL (2006) Frequent finding of the androgen receptor A645D variant in normal population. The Journal of clinical endocrinology and metabolism 91: 3228-3231.

84. Huddart RA, Norman A, Moynihan C, Horwich A, Parker C, et al. (2005) Fertility, gonadal and sexual function in survivors of testicular cancer. British journal of cancer 93: 200-207.

85. Alvarez NR, Lee TM, Solorzano CC (2005) Complete androgen insensitivity syndrome: the role of the endocrine surgeon. The American surgeon 71: 241-243.

86. Stovall M, Donaldson SS, Weathers RE, Robison LL, Mertens AC, et al. (2004) Genetic effects of radiotherapy for childhood cancer: gonadal dose reconstruction. International journal of radiation oncology, biology, physics 60: 542-552.

87. Agarwal A, Ranganathan P, Kattal N, Pasqualotto F, Hallak J, et al. (2004) Fertility after cancer: a prospective review of assisted reproductive outcome with banked semen specimens. Fertility and sterility 81: 342-348.

88. Ragni G, Somigliana E, Restelli L, Salvi R, Arnoldi M, et al. (2003) Sperm banking and rate of assisted reproduction treatment: insights from a 15-year cryopreservation program for male cancer patients. Cancer 97: 1624-1629.

89. Kawai K (2002) [Long-term side effects of chemotherapy for testicular cancer]. Gan to kagaku ryoho Cancer & chemotherapy 29: 1300-1305. In Japanese.

90. Vaughn DJ, Gignac GA, Meadows AT (2002) Long-term medical care of testicular cancer survivors. Annals of internal medicine 136: 463-470.

91. Kobayashi H, Larson K, Sharma RK, Nelson DR, Evenson DP, et al. (2001) DNA damage in patients with untreated cancer as measured by the sperm chromatin structure assay. Fertility and sterility 75: 469-475.

92. Engeler DS, Hosli PO, John H, Bannwart F, Sulser T, et al. (2000) Early orchiopexy: prepubertal intratubular germ cell neoplasia and fertility outcome. Urology 56: 144-148.

93. Tekin A, Aygun YC, Aki FT, Ozen H (2000) Bilateral germ cell cancer of the testis: a report of 11 patients with a long-term follow-up. BJU international 85: 864-868.

94. Petersen PM, Hansen SW (1999) The course of long-term toxicity in patients treated with cisplatin-based chemotherapy for non-seminomatous germ-cell cancer. Annals of oncology : official journal of the European Society for Medical Oncology / ESMO 10: 1475-1483.

95. Sedlmayer F, Joos H, Deutschmann H, Rahim H, Merz F, et al. (1999) [Long-term tumor control and fertility after para-aortic limited radiotherapy of stage I seminoma]. Strahlentherapie und Onkologie : Organ der Deutschen Rontgengesellschaft [et al] 175: 320-324. In German.

96. Gruber G, Schwegler N (1999) [The gonadal loading during the irradiation of lymph outflow in operated seminomas. In-vivo dosimetry]. Strahlentherapie und Onkologie : Organ der Deutschen Rontgengesellschaft [et al] 175: 185-189. In German.

97. Ganem JP, Workman KR, Shaban SF (1999) Testicular microlithiasis is associated with testicular pathology. Urology 53: 209-213.

98. Naysmith TE, Blake DA, Harvey VJ, Johnson NP (1998) Do men undergoing sterilizing cancer treatments have a fertile future? Human reproduction (Oxford, England) 13: 3250-3255.

99. Raimoldi A, Berti GL, Canclini L, Giola V, Leidi GL, et al. (1997) [Papillary cystadenoma of the epididymis. 2 case reports]. Archivio italiano di urologia, andrologia : organo ufficiale [di] Societa italiana di ecografia urologica e nefrologica / Associazione ricerche in urologia 69: 309-311. In Italian.

100. Herr HW, Bar-Chama N, O'Sullivan M, Sogani PC (1998) Paternity in men with stage I testis tumors on surveillance. Journal of clinical oncology : official journal of the American Society of Clinical Oncology 16: 733-734.

101. Herrmann T (1997) [Radiation reactions in the gonads: importance in patient counseling]. Strahlentherapie und Onkologie : Organ der Deutschen Rontgengesellschaft [et al] 173: 493-501. In German.

102. Giwercman A, Thomsen JK, Hertz J, Berthelsen JG, Jensen V, et al. (1997) Prevalence of carcinoma in situ of the testis in 207 oligozoospermic men from infertile couples: prospective study of testicular biopsies. BMJ (Clinical research ed ) 315: 989-991.

103. Baniel J, Roth BJ, Foster RS, Donohue JP (1996) Cost- and risk-benefit considerations in the management of clinical stage I nonseminomatous testicular tumors. Annals of surgical oncology 3: 86-93.

104. Houlgatte A, Houdelette P, Berlizot P, Fournier R, Bernard O, et al. (1995) [Bilateral tumors of the testis: the role of the diagnosis of carcinoma in situ in early detection]. Progres en urologie : journal de l'Association francaise d'urologie et de la Societe francaise d'urologie 5: 540-543. In French.

105. Stephenson WT, Poirier SM, Rubin L, Einhorn LH (1995) Evaluation of reproductive capacity in germ cell tumor patients following treatment with cisplatin, etoposide, and bleomycin. Journal of clinical oncology : official journal of the American Society of Clinical Oncology 13: 2278-2280.

106. Baniel J, Roth BJ, Foster RS, Donohue JP (1995) Cost and risk benefit in the management of clinical stage II nonseminomatous testicular tumors. Cancer 75: 2897-2903.

107. Pacini F, Gasperi M, Fugazzola L, Ceccarelli C, Lippi F, et al. (1994) Testicular function in patients with differentiated thyroid carcinoma treated with radioiodine. Journal of nuclear medicine : official publication, Society of Nuclear Medicine 35: 1418-1422.

108. Petersen PM, Hansen SW, Giwercman A, Rorth M, Skakkebaek NE (1994) Dose-dependent impairment of testicular function in patients treated with cisplatin-based chemotherapy for germ cell cancer. Annals of oncology : official journal of the European Society for Medical Oncology / ESMO 5: 355-358.

109. Bohm E, Walz PH, Tan KH (1993) [A combination of spermatocytic and classic seminoma, mature teratoma and carcinoma in situ of the testis. An attempt at an etiologic explanation]. Zentralblatt fur Pathologie 139: 255-260.

110. de Bruin MJ, Oosterhof GO, Debruyne FM (1993) Nerve-sparing retroperitoneal lymphadenectomy for low stage testicular cancer. British journal of urology 71: 336-339.

111. Lahdenne P (1992) Late sequelae of gonadal, mediastinal and oral teratomas in childhood. Acta paediatrica (Oslo, Norway : 1992) 81: 235-238.

112. Charak BS, Gupta R, Mandrekar P, Sheth NA, Banavali SD, et al. (1990) Testicular dysfunction after cyclophosphamide-vincristine-procarbazine-prednisolone chemotherapy for advanced Hodgkin's disease. A long-term follow-up study. Cancer 65: 1903-1906.

113. Holland-Moritz H, Krause W (1990) [Use of sperm cryopreservation by tumor patients]. Der Hautarzt; Zeitschrift fur Dermatologie, Venerologie, und verwandte Gebiete 41: 204-206.

114. Barth V (1990) [Retrograde ejaculation as a cause of aspermia following retroperitoneal lymph node excision and the effective use of alpha sympathomimetic drugs]. Zeitschrift fur Urologie und Nephrologie 83: 115-119.

115. Kreuser ED, Kurrle E, Hetzel WD, Heymer B, Porzsolt F, et al. (1989) [Reversible germ cell toxicity following aggressive chemotherapy in patients with testicular tumors: results of a prospective study]. Klinische Wochenschrift 67: 367-378.

116. Schubert J, Held HJ, Kelly LU, Tolkendorf E (1989) [Autologous frozen sperm--an alternative in infertility in the patient with invasively treated testicular tumor?]. Zeitschrift fur Urologie und Nephrologie 82: 209-216.

117. Bouchot O, Plougastel ML, Karam G, Bochereau G, Auvigne J (1989) [Sterility and tumors of the testis. Study of late exocrine and endocrine functions in stage I or IIA tumors]. Journal d'urologie 95: 367-371.

118. Burke AP, Mostofi FK (1988) Intratubular malignant germ cells in testicular biopsies: clinical course and identification by staining for placental alkaline phosphatase. Modern pathology : an official journal of the United States and Canadian Academy of Pathology, Inc 1: 475-479.

119. Carroll PR, Morse MJ, Whitmore WF, Sogani PC, Klotz L, et al. (1987) Fertility status of patients with clinical stage I testis tumors on a surveillance protocol. The Journal of urology 138: 70-72.

120. Nijman JM, Schraffordt Koops H, Kremer J, Sleijfer DT (1987) Gonadal function after surgery and chemotherapy in men with stage II and III nonseminomatous testicular tumors. Journal of clinical oncology : official journal of the American Society of Clinical Oncology 5: 651-656.

121. Kreuser ED, Harsch U, Hetzel WD, Schreml W (1986) Chronic gonadal toxicity in patients with testicular cancer after chemotherapy. European journal of cancer & clinical oncology 22: 289-294.

122. Schover LR, Gonzales M, von Eschenbach AC (1986) Sexual and marital relationships after radiotherapy for seminoma. Urology 27: 117-123.

123. Rieker PP, Edbril SD, Garnick MB (1985) Curative testis cancer therapy: psychosocial sequelae. Journal of clinical oncology : official journal of the American Society of Clinical Oncology 3: 1117-1126.

124. Leary FJ, Resseguie LJ, Kurland LT, O'Brien PC, Emslander RF, et al. (1984) Males exposed in utero to diethylstilbestrol. JAMA : the journal of the American Medical Association 252: 2984-2989.

125. Foulques H, Timbal Y, Jardin A (1984) [Bilateral testicular cancer of different histological types]. Journal d'urologie 90: 369-374.

126. Schein PS, Winokur SH (1975) Immunosuppressive and cytotoxic chemotherapy: long-term complications. Annals of internal medicine 82: 84-95.

127. Richiardi L, Akre O, Montgomery SM, Lambe M, Kvist U, et al. (2004) Fecundity and twinning rates as measures of fertility before diagnosis of germ-cell testicular cancer. Journal of the National Cancer Institute 96: 145-147.

128. Fossa SD, Kravdal O (2000) Fertility in Norwegian testicular cancer patients. British journal of cancer 82: 737-741.

129. Jacobsen R, Bostofte E, Engholm G, Hansen J, Skakkebaek NE, et al. (2000) Fertility and offspring sex ratio of men who develop testicular cancer: a record linkage study. Human reproduction (Oxford, England) 15: 1958-1961.

130. Petersen PM, Skakkebaek NE, Vistisen K, Rorth M, Giwercman A (1999) Semen quality and reproductive hormones before orchiectomy in men with testicular cancer. Journal of clinical oncology : official journal of the American Society of Clinical Oncology 17: 941-947.

131. Moller H (1998) Trends in sex-ratio, testicular cancer and male reproductive hazards: are they connected? APMIS : acta pathologica, microbiologica, et immunologica Scandinavica 106: 232-238; discussion 238-239.

132. Heimdal K, Olsson H, Tretli S, Flodgren P, Borresen AL, et al. (1996) Risk of cancer in relatives of testicular cancer patients. British journal of cancer 73: 970-973.

133. Forman D, Pike M, Davey G, Dawson S, Baker K, et al. (1994) Aetiology of testicular cancer: association with congenital abnormalities, age at puberty, infertility, and exercise. United Kingdom Testicular Cancer Study Group. BMJ (Clinical research ed ) 308: 1393-1399.

134. Haughey BP, Graham S, Brasure J, Zielezny M, Sufrin G, et al. (1989) The epidemiology of testicular cancer in upstate New York. American journal of epidemiology 130: 25-36.

135. Gershman ST, Stolley PD (1988) A case-control study of testicular cancer using Connecticut tumour registry data. International journal of epidemiology 17: 738-742.

136. Brown LM, Pottern LM, Hoover RN (1987) Testicular cancer in young men: the search for causes of the epidemic increase in the United States. Journal of epidemiology and community health 41: 349-354.

137. Depue RH, Pike MC, Henderson BE (1983) Estrogen exposure during gestation and risk of testicular cancer. Journal of the National Cancer Institute 71: 1151-1155.

138. Henderson BE, Benton B, Jing J, Yu MC, Pike MC (1979) Risk factors for cancer of the testis in young men. International journal of cancer Journal international du cancer 23: 598-602.

139. coldman AJ, Elwood JM, Gallagher RP (1982) Sports activity and risk of testicular cancer. BritJCancer 46: 749-756.
